# Supplementary material for: Resource Screening and Inheritance Analysis of Fusarium oxysporum sp. conglutinans Race 2 Resistance in Cabbage (Brassica oleracea var. capitata)
Source: Genes (Basel). 2022 Sep 4;13(9):1590. doi: 10.3390/genes13091590 (PMC9498596; doi:10.3390/genes13091590)
Supplement: Supplementary file 1 [file genes-13-01590-s001.zip › genes-1874803-supplementary.pdf]

Table S1. Detailed information and resistance evaluation of 166 tested cabbage accessions.

Code	Accession name	Origin	Season	,Maturity	Head shape	Leaf color	DI value of race 1	Resistance level of race 1	DI value of race 2	Resistance level of race 2
FW1	BI-16	USA	Autumn	Mid-early	Round	Gray	0.00±0.00	HR	0.00±0.00	HR
FW2	96-100	India	Autumn	Early	Round	Gray green	0.00±0.00	HR	81.33±2.31	HS
FW3	01-20	Canada	Spring	Early	Round	Green	92.00±4.00	HS	86.67±2.31	HS
FW4	20-2-5	China	Autumn	Late	Flat	Gray green	42.67±2.31	MR	100.00±0.00	HS
FW5	23202	China	Autumn	Late	Flat	Gray green	89.33±6.11	HS	100.00±0.00	HS
FW6	8020	China	Autumn	Mid-late	Flat	Gray green	80.00±6.93	HS	100.00±0.00	HS
FW7	8282	Japan	Autumn	Mid-late	Flat	Green	69.33±2.31	S	100.00±0.00	HS
FW8	21-3	China	Autumn	Mid-late	Flat	Gray green	100.00±0.00	HS	100.00±0.00	HS
FW9	2-18-1	China	Autumn	Late	Flat	Green	100.00±0.00	HS	100.00±0.00	HS
FW10	XF	Taiwan, China	Autumn	Mid-late	Flat	Gray green	58.67±2.31	S	50.67±6.11	S
FW11	T41	Taiwan, China	Autumn	Mid-late	Flat	Dark green	45.33±6.11	MR	82.67±6.11	HS
FW12	QD	Japan	Autumn	Late	Flat	Gray	0.00±0.00	HR	0.00±0.00	HR
FW13	24-5-3	China	Autumn	Late	Flat	Green	24.00±0.00	R	40.00±0.00	MR
FW14	MD	Japan	Autumn	Late	Flat	Gray green	0.00±0.00	HR	0.00±0.00	HR
FW15	XJ253	Japan	Autumn	Mid-late	Flat	Dark green	85.67±6.03	HS	81.33±4.62	HS
FW16	SLS288	Japan	Autumn	Late	Flat	Dark green	85.33±2.31	HS	100.00±0.00	HS
FW17	MWT310	Japan	Autumn	Late	Flat	Dark green	0.00±0.00	HR	0.00±0.00	HR
FW18	YF	Japan	Overwintering	Medium	Round	Green	0.00±0.00	HR	86.67±2.31	HR
FW19	PT	Netherlands	Overwintering	Early	Round	Dark green	93.33±6.11	HS	98.67±2.31	HS
FW20	1039	Netherlands	Overwintering	Medium	Round	Dark green	48.00±6.93	MR	38.67±6.11	MR
FW21	HN7	Korea	Autumn	Early	Round	Green	47.00±1.73	MR	50.67±2.31	S
FW22	T4	Korea	Spring	Early	Round	Dark green	65.33±2.31	S	82.67±6.11	HS
FW23	ZQ	Japan	Autumn	Early	Round	Gray green	0.00±0.00	HR	0.00±0.00	HR
FW24	96-109	Denmark	Autumn	Mid-early	Round	Dark green	50.00±2.00	S	42.67±2.31	MR
FW25	XQ	Korea	Autumn	Mid-early	Round	Gray	0.00±0.00	HR	0.00±0.00	HR
FW26	CB201	Thailand	Autumn	Early	Round	Dark green	84.33±4.04	HS	58.67±6.11	S
FW27	LQDH	Korea	Autumn	Early	Round	Green	78.33±2.89	HS	95.00±8.66	HS
FW28	DH811	Netherlands	Autumn	Mid-early	Round	Green	70.67±6.11	HS	100.00±0.00	HS
FW29	Globe star	Japan	Autumn	Early	Round	Gray green	57.11±5.75	S	84.00±4.00	HS
FW30	084	Denmark	Spring	Early	Round	Gray	100.00±0.00	HS	97.33±4.62	HS
FW31	79-156	Denmark	Spring	Early	Round	Gray green	38.67±2.31	MR	41.33±5.51	MR
FW32	SG643	Netherlands	Spring	Early	Round	Green	46.67±2.31	MR	77.33±2.31	HS
FW33	SJMT	Japan	Spring	Early	Round	Green	0.00±0.00	HR	46.67±2.31	MR
FW34	Y162	Netherlands	Spring	Medium	Round	Gray green	0.00±0.00	HR	13.33±2.31	R
FW35	Y165	Russia	Spring	Early	Round	Green	94.44±5.09	HS	100.00±0.00	HS
FW36	650L	Netherlands	Spring	Medium	Round	Gray green	0.00±0.00	HR	6.67±2.31	HR

FW37	MWZS	Japan	Spring	Mid-early	Round	Gray green	50.67±4.04	S	62.67±6.11	S
FW38	Nball	USA	Spring	Mid-early	Round	Gray green	92.00±4.00	HS	97.33±4.62	HS
FW39	BNZS	China	Spring	Medium	Round	Green	50.67±2.31	S	81.33±2.31	HS
FW40	PD	USA	Spring	Mid-early	Round	Gray green	65.33±2.31	S	66.67±4.62	S
FW41	JS	Bulgaria	Autumn	Mid-early	Round	Gray green	12.00±4.00	R	44.00±4.00	MR
FW42	MGZGL	USA	Spring	Early	Round	Green	0.00±0.00	HR	46.67±2.31	MR
FW43	HY	Japan	Autumn	Mid-late	Flat	Gray green	46.00±1.73	MR	54.00±1.73	S
FW44	Ikama266	Japan	Autumn	Mid-late	Flat	Green	50.67±2.31	S	41.33±2.31	MR
FW45	AQN266	Japan	Autumn	Late	Flat	Gray green	0.00±0.00	HR	6.67±2.31	HR
FW46	HT	Japan	Autumn	Mid-late	Flat	Dark green	0.00±0.00	HR	6.67±2.31	HR
FW47	YRHD	Japan	Autumn	Mid-late	Flat	Gray green	0.00±0.00	HR	8.33±2.89	HR
FW48	HN1	Japan	Autumn	Late	Flat	Dark green	0.00±0.00	HR	4.00±0.00	HR
FW49	YRRGGL	Japan	Autumn	Mid-late	Flat	Green	41.33±2.31	MR	48.00±4.00	MR
FW50	YC	Japan	Autumn	Late	Flat	Green	50.67=2.31	S	85.33±2.31	HS
FW51	Gunma	USA	Autumn	Late	Flat	Green	42.44±2.14	MR	58.33±2.89	S
FW52	YF	Japan	Autumn	Late	Flat	Green	24.00±4.00	R	64.00±4.00	S
FW53	QG384-1	Japan	Autumn	Late	Flat	Dark green	77.33±6.11	HS	94.33±2.08	HS
FW54	HB1	Japan	Autumn	Late	Flat	Green	26.67±2.31	R	17.33±2.31	R
FW55	HB2F6	Japan	Autumn	Late	Flat	Green	54.44±5.04	S	90.00±10.00	HS
FW56	XXY	Japan	Autumn	Late	Flat	Green	20.00±4.00	R	33.33±5.77	MR
FW57	CLF6	Japan	Autumn	Late	Flat	Gray green	30.67±2.31	MR	13.33±2.31	R
FW58	HB34	Japan	Autumn	Mid-late	Flat	Dark green	0.00±0.00	HR	42.67±2.31	HR
FW59	DNM	China	Autumn	Late	Flat	Green	0.00±0.00	HR	10.67±2.31	R
FW60	OZYQ	Netherlands	Overwintering	Late	Round	Green	88.00±4.00	HS	100.00±0.00	HS
FW61	HB1186	Japan	Autumn	Mid-early	Round	Green	0.00±0.00	HR	0.00±0.00	HR
FW62	JB	Japan	Autumn	Mid-early	Round	Green	8.00±0.00	HR	17.33±2.31	R
FW63	TCGL	China	Autumn	Late	Flat	Green	22.67±2.31	R	63.33±5.77	S
FW64	YD2	Japan	Overwintering	Late	Flat	Dark green	56.00±4.00	S	89.33±2.31	HS
FW65	HN25	Japan	Overwintering	Late	Flat	Dark green	43.33±2.89	MR	81.67±5.77	HS
FW66	308DS	Japan	Overwintering	Late	Flat	Gray	4.00±6.93	HR	0.00±0.00	HR
FW67	JLL	Japan	Overwintering	Late	Flat	Green	20.00±4.00	R	34.67±2.31	MR
FW68	HY26	Japan	Overwintering	Late	Flat	Gray green	36.00±0.00	MR	34.67±4.62	MR
FW69	YU1	Japan	Overwintering	Late	Flat	Green	26.67±2.31	R	6.67±2.31	HR
FW70	11-62	Japan	Overwintering	Late	Flat	Gray	84.00±6.93	HS	84.00±2.31	HS
FW71	HJJ	Japan	Overwintering	Late	Flat	Dark green	0.00±0.00	HR	13.33±6.11	R
FW72	YQ	Japan	Overwintering	Late	Flat	Dark green	44.00±4.00	MR	64.00=6.93	S
FW73	KBK	Japan	Overwintering	Late	Flat	Gray green	53.33±2.31	S	44.00±4.00	MR
FW74	YK143	Japan	Overwintering	Late	Round	Green	97.33±2.31	HS	77.33±6.11	HS
FW75	E05-13	Russia	Overwintering	Late	Round	Green	57.33±4.62	S	100.00±0.00	HS
FW76	E05-17	Russia	Overwintering	Late	Round	Dark green	68.00±0.00	S	78.33±5.77	HS
FW77	H4	Japan	Overwintering	Late	Flat	Green	10.00±3.33	R	13.33±2.89	R
FW78	1038	Netherlands	Overwintering	Medium	Round	Green	0.00±0.00	HR	12.78±2.54	R
FW79	YMS	Netherlands	Overwintering	Late	Round	Gray green	0.00±0.00	HR	0.00±0.00	HR

FW80	HB48	Japan	Overwintering	Late	Flat	Dark green	9.33±4.62	HR	89.33±2.31	HR
FW81	10-511	Netherlands	Overwintering	Late	Round	Gray green	54.67±2.31	S	97.33±4.62	HS
FW82	H2	China	Spring	Mid-early	Round	Gray green	46.67±6.11	MR	64.33±0.58	S
FW83	A221	Japan	Overwintering	Mid-early	Round	Dark green	69.33±2.31	S	86.67±2.31	HS
FW84	PL	Korea	Autumn	Mid-early	Round	Green	44.00±4.00	MR	58.66±2.31	S
FW85	JB	Japan	Autumn	Mid-early	Round	Gray green	22.67±2.31	R	44.00±4.00	MR
FW86	YC205	Korea	Autumn	Early	Round	Gray green	0.00±0.00	HR	6.67±2.31	HR
FW87	YC207	Korea	Autumn	Early	Round	Green	0.00±0.00	HR	6.67±2.31	HR
FW88	XT0102	Korea	Autumn	Early	Round	Green	36.00±0.00	MR	49.33±4.62	MR
FW89	bejo1012	Netherlands	Autumn	Early	Round	Gray	0.00±0.00	HR	11.67±2.89	R
FW90	YDWW	Japan	Autumn	Early	Round	Green	74.67±4.62	HS	65.33±8.33	S
FW91	Y159	Netherlands	Spring	Early	Round	Gray green	50.00±2.00	S	53.67±7.77	S
FW92	JTM	Netherlands	Spring	Early	Round	Green	0.00±0.00	HR	93.33±6.11	HS
FW93	Y181	Netherlands	Spring	Early	Round	Green	45.33±2.31	MR	27.33±3.06	R
FW94	MNK	Netherlands	Spring	Early	Round	Gray green	63.33±5.77	S	93.33±11.55	HS
FW95	YOP	Italia	Spring	Early	Round	Green	53.33±4.62	S	52.00±4.00	S
FW96	TZD45A	Thailand	Spring	Early	Round	Gray green	52.00±0.00	S	77.33±2.31	HS
FW97	LY	Japan	Autumn	Early	Round	Green	0.00±0.00	HR	6.67±2.31	HR
FW98	BUSOMI	Japan	Autumn	Mid-early	Round	Gray green	77.33±2.31	HS	76.67±2.89	HS
FW99	lion	Japan	Autumn	Mid-early	Round	Gray green	0.00±0.00	HR	4.00±0.00	HR
FW100	JSGL	Japan	Autumn	Mid-early	Round	Green	96.00±0.00	HS	100.00±0.00	HS
FW101	Jersy Queen	USA	Autumn	Late	Flat	Green	38.67±2.31	MR	70.00±2.00	HS
FW102	BS	USA	Autumn	Late	Flat	Green	0.00±0.00	HR	8.89±3.85	HR
FW103	LT	USA	Autumn	Late	Flat	Green	0.00±0.00	HR	4.67±0.58	HR
FW104	WS	USA	Autumn	Late	Flat	Green	0.00±0.00	HR	4.00±0.00	HR
FW105	MYBQ	China	Autumn	Late	Flat	Gray green	20.00±4.00	R	35.67±4.04	MR
FW106	HN2014Q160	Japan	Autumn	Late	Flat	Gray green	0.00±0.00	HR	6.67±2.31	HR
FW107	HN2014Q494	Japan	Autumn	Late	Flat	Gray green	48.00±4.00	MR	100.00±0.00	HS
FW108	HN2014Q495	Japan	Autumn	Late	Flat	Gray green	70.67±2.31	HS	90.67±9.24	HS
FW109	STK	Netherlands	Autumn	Late	Round	Gray green	13.33±2.31	R	30.67±2.31	MR
FW110	XY6	China	Autumn	Late	Flat	Green	36.00±0.00	MR	61.33±4.62	S
FW111	070	Japan	Autumn	Late	Flat	Gray green	0.00±0.00	HR	4.00±0.00	HR
FW112	044	Japan	Autumn	Late	Flat	Green	53.33±2.31	S	57.33±8.33	S
FW113	JPQ2	China	Autumn	Late	Flat	Dark green	55.67±4.04	S	86.67±2.31	HS
FW114	JLGL	Japan	Autumn	Early	Round	Dark green	0.00±0.00	HR	0.00±0.00	HR
FW115	BJGL	Japan	Autumn	Early	Round	Green	0.00±0.00	HR	17.33±2.31	R
FW116	MYF	Japan	Autumn	Early	Round	Gray green	12.00±0.00	R	80.00±4.00	HS
FW117	JDNLB	Japan	Autumn	Early	Round	Dark green	0.00±0.00	HR	6.67±2.31	HR
FW118	HNBG08	Japan	Autumn	Early	Round	Gray green	26.67±2.31	R	8.00±0.00	HR
FW119	HNBG16	Japan	Autumn	Early	Round	Green	0.00±0.00	HR	8.00±0.00	HR
FW120	HNBG18	Japan	Autumn	Early	Round	Gray green	0.00±0.00	HR	14.67±2.31	R
FW121	CF3	China	Autumn	Early	Round	Green	37.33±4.62	MR	78.67±4.62	HS
FW122	SZQ	Japan	Autumn	Early	Round	Gray green	0.00±0.00	HR	4.00±0.00	HR

FW123	2011Q9-1	Japan	Autumn	Mid-early	Round	Green	58.67±2.31	S	71.67±7.64	HS
FW124	2011Q16-1	Japan	Autumn	Mid-early	Round	Green	61.33±2.31	S	80.33±4.51	HS
FW125	2011Q32-1	Japan	Autumn	Early	Round	Dark green	0.00±0.00	HR	70.67±2.31	HS
FW126	2011Q53-1	Japan	Autumn	Late	Round	Green	56.00±4.00	S	86.67±2.31	HS
FW127	07C1493	Japan	Autumn	Mid-early	Round	Green	0.00±0.00	HR	6.67±2.31	HR
FW128	T100O	Switzerland	Autumn	Early	Round	Dark green	63.33±5.77	S	94.67±4.62	HS
FW129	CH-750	Japan	Autumn	Mid-early	Round	Dark green	37.33±2.31	MR	14.67±2.31	R
FW130	894	Japan	Autumn	Mid-early	Round	Green	0.00±0.00	HR	6.67±2.31	HR
FW131	HC70-1	Japan	Autumn	Mid-early	Round	Gray green	0.00±0.00	HR	25.33±9.24	R
FW132	HBG-1	Japan	Autumn	Early	Round	Green	13.33±2.31	R	21.33±2.31	R
FW133	PPF-2	Japan	Autumn	Early	Round	Dark green	24.00±0.00	R	37.33±4.62	MR
FW134	ML-2	Japan	Autumn	Early	Round	Gray green	0.00±0.00	HR	6.67±2.31	HR
FW135	01-88	Canada	Spring	Early	Round	Green	100.00±0.00	HS	100.00±0.00	HS
FW136	ST-4	Korea	Spring	Early	Round	Dark green	0.00±0.00	HR	4.00±0.00	HR
FW137	SCA026	Korea	Spring	Early	Round	Gray green	45.33±2.31	MR	96.00±0.00	HS
FW138	XLN	Japan	Spring	Early	Round	Dark green	69.33±2.31	HR	96.00±4.00	HS
FW139	YFJZ	Japan	Spring	Early	Round	Dark green	80.00±0.00	HS	100.00±0.00	HS
FW140	JZP	Japan	Spring	Early	Round	Gray green	44.00±0.00	MR	98.67±2.31	HS
FW141	11C2425	Japan	Spring	Medium	Round	Dark green	0.00±0.00	HR	13.33±2.89	HR
FW142	DC-06	Japan	Spring	Mid-early	Round	Dark green	38.67±2.31	MR	48.00±4.00	MR
FW143	TT8	Netherlands	Spring	Medium	Round	Gray green	37.78±3.85	MR	62.22±3.85	S
FW144	FT	Switzerland	Spring	Early	Round	Green	33.33±2.31	MR	48.00±4.00	MR
FW145	NC89	Japan	Spring	Early	Round	Dark green	0.00±0.00	HR	5.00±0.00	HR
FW146	N139	Japan	Spring	Mid-early	Round	Green	35.00±0.00	MR	30.67±6.11	MR
FW147	07C1494	Japan	Spring	Medium	Round	Green	20.00±0.00	R	27.33±4.04	R
FW148	KAG733	Japan	Spring	Mid-early	Round	Gray green	66.67±2.31	S	69.33±6.11	S
FW149	Puma	Netherlands	Spring	Mid-early	Round	Green	88.00±6.93	HS	100.00±0.00	HS
FW150	Charmant	Japan	Spring	Mid-early	Round	Gray green	70.67±5.69	HS	76.00±6.93	HS
FW151	Ferry round dutch	Netherlands	Spring	Mid-early	Round	Green	96.00±6.93	HS	4.00±0.00	HS
FW152	HNBG17	Japan	Spring	Mid-early	Round	Dark green	0.00±0.00	HR	6.67±2.31	HR
FW153	HNBG19	Japan	Spring	Mid-early	Round	Dark green	30.67±2.31	MR	84.00±4.00	HS
FW154	CGZ	Japan	Spring	Early	Round	Dark green	33.33±6.11	MR	62.67±8.33	S
FW155	XJH	China	Spring	Early	Round	Green	100.00±0.00	HS	98.67±2.31	HS
FW156	HC1	Japan	Overwintering	Mid-early	Round	Dark green	0.00±0.00	HR	85.33±2.31	HR
FW157	107GL	Japan	Overwintering	Mid-early	Round	Gray green	0.00±0.00	HR	85.33±2.31	HR
FW158	ML	Japan	Overwintering	Mid-early	Round	Gray green	0.00±0.00	HR	4.00±0.00	HR
FW159	BMT620	Japan	Overwintering	Mid-early	Round	Gray green	45.33±4.62	MR	22.67±2.31	R
FW160	JQ90	Japan	Overwintering	Early	Round	Gray green	0.00±0.00	HR	4.00±0.00	HR
FW161	1186	Japan	Overwintering	Mid-early	Round	Gray green	29.33±2.31	R	62.22±3.85	S
FW162	A1012	Japan	Overwintering	Mid-early	Round	Green	0.00±0.00	HR	89.33±2.31	HR
FW163	SH101	USA	Overwintering	Late	Flat	Gray green	12.00±0.00	R	22.67±2.31	R
FW164	RG22	China	Overwintering	Late	Flat	Gray green	17.33±2.31	R	6.67±2.31	HR
FW165	JY82	Japan	Overwintering	Early	Flat	Gray green	37.33±2.31	MR	22.67±2.31	R

FW166	H137	Japan	Spring	Mid-early	Round	Dark green	16.00±0.00	R	56.00±4.00	S

Note: Disease Index data are mean ±standard deviation.
